# The role of s-palmitoylation in neurological diseases: implication for zDHHC family

**DOI:** 10.3389/fphar.2023.1342830

**Published:** 2024-01-16

**Authors:** Dan Liao, Yutao Huang, Dan Liu, Haofuzi Zhang, Xinyu Shi, Xin Li, Peng Luo

**Affiliations:** ^1^ Department of Neurosurgery, Xijing Hospital, Fourth Military Medical University, Xi’an, China; ^2^ School of Life Science, Northwest University, Xi’an, China; ^3^ Department of Anesthesiology, Xijing Hospital, Fourth Military Medical University, Xi’an, China

**Keywords:** S-palmitoylation, protein acyltransferase, zDHHC, glutamate receptor, scaffold protein, neurological diseases

## Abstract

S-palmitoylation is a reversible posttranslational modification, and the palmitoylation reaction in human-derived cells is mediated by the zDHHC family, which is composed of S-acyltransferase enzymes that possess the DHHC (Asp-His-His-Cys) structural domain. zDHHC proteins form an autoacylation intermediate, which then attaches the fatty acid to cysteine a residue in the target protein. zDHHC proteins sublocalize in different neuronal structures and exert dif-ferential effects on neurons. In humans, many zDHHC proteins are closely related to human neu-rological disor-ders. This review focuses on a variety of neurological disorders, such as AD (Alz-heimer’s disease), HD (Huntington’s disease), SCZ (schizophrenia), XLID (X-linked intellectual disability), attention deficit hyperactivity disorder and glioma. In this paper, we will discuss and summarize the research progress regarding the role of zDHHC proteins in these neu-rological disorders.

## 1 Introduction

Protein S-acylation is the posttranslational modification of proteins by long-chain fatty acids with different carbon contents, such as myristic acid (C14:0), palmitic acid (C16:0), palmitoleic acid (C16:1), stearic acid (C18:0), oleic acid (C18:1), arachidonic acid (C20:4), and eicosapentaenoic acid (C20:5) (Nuskova et al., 2023). Among them, palmitoleic acid (C16:1) modifications are very common, so protein S-acylation is also commonly referred to as protein palmitoylation (De and Sadhukhan, 2018). Palmitoylation was first reported in 1979 (Schmidt and Schlesinger, 1979), and at least 3,500 proteins are known to be palmitoylated at more than 9,000 palmitoylation sites (Zhou et al., 2019). There are three main functions of palmitoylation. First, palmitoylation increases the hydrophobicity of a protein, leading to changes in conformation, stability, localization, and binding affinity to cofactors (Yang et al., 2020). Second, palmitoylation ensures structural integrity and the proper folding of proteins and restricts faulty protein synthesis and ubiquitination-mediated protein degradation (Wang et al., 2020). Finally, palmitoylation affects the transport of membrane-associated proteins from early secretory sites to appropriate cellular destinations by regulating protein interactions (Hao et al., 2020).

Traditionally, palmitoylation can be classified into N-palmitoylation, O-palmitoylation, and S-palmitoylation depending on the site of modification. However, it is worth noting that of the three modifications, only S-palmitoylation is a reversible, dynamically regulated posttranslational modification that can be completed in seconds to hours, while N- and O-palmitoylation are irreversible (Rocks et al., 2010). Since S-palmitoylation occurs more often due to its reversible nature, protein palmitoylation is commonly refers to S-palmitoylation (Das et al., 2021). The S-palmitoylation reaction in eukaryotic cells is mediated by PATs (protein acyltransferases). These PATs usually contain conserved DHHC (aspartate-histidine-histidine-cysteine) tetrapeptides and zinc finger structural domains and are therefore defined as proteins belonging to the zDHHC family (Lemonidis et al., 2015). Over the past few years, the relationship between zDHHC family proteins and neurological disorders, including intellectual disability, HD (Huntington’s disease) and (SCZ) schizophrenia, has been explored by using cell culture, animal models, and zDHHC-deficient mice (Jin et al., 2021). In this review, we will summarize recent findings on the role of zDDHC in neurological diseases and discuss the therapeutic potential of compounds targeting zDHHC activity.

## 2 The zDHHC family of protein acyltransferases

### 2.1 The discovery of zDHHC proteins

Originally, palmitate was found to be present in cells as CoA (palmitoyl-coenzyme A). However, the concentration of free unbound acyl-CoA was not sufficient for spontaneous S-palmitoylation (Faergeman and Knudsen, 1997). Therefore, researchers have speculated that the palmitoylation of proteins mainly relies on the action of enzymes. To support this view, Erf2p, an enzyme catalyzing cysteine acyl transfer to Ras2, was discovered in 2002 and was defined as the first PAT (Lobo et al., 2002). Subsequently, a series of PATs, including HIP14 (Huntington-interacting proteins 14) (Lemarie et al., 2021), SERZ-beta (Sertoli cell gene with a zinc finger domain-beta) (Fang et al., 2006), and HIP14L (Huntingtin-interacting protein 14-like) (Lemarie et al., 2021), have been reported to play a catalytic role in palmitoylation. Finally, it was concluded that palmitoylation is catalyzed by low-abundance, polytopic eukaryotic integral membrane PATs (Rana et al., 2019).

Structurally, it was found that the common feature among PATs is a highly conserved structural domain containing DHHC (Rocks et al., 2010). This is why some articles refer to PATs as DHHC proteins (Ladygina et al., 2011). However, this DHHC domain also coordinates the folding of the two Zn^2+^ atoms in the zinc finger structural domain, which is essential for the structural stability of PATs (Gonzalez Montoro et al., 2013). Therefore, more authors have classified the family of PATs containing DHHC structural domains as zDHHC, and this nomenclature is used throughout the remainder of this review. At present, there are 23 different zDHHC isoforms in the mouse and human proteome databases, named zDHHC1 to zDHHC24, skipping zDHHC10. All of the zDHHC proteins exhibit different self-acylation abilities and different levels of catalytic efficiency (Malgapo and Linder, 2021).

### 2.2 The structure of zDHHC proteins

**FIGURE 1:**
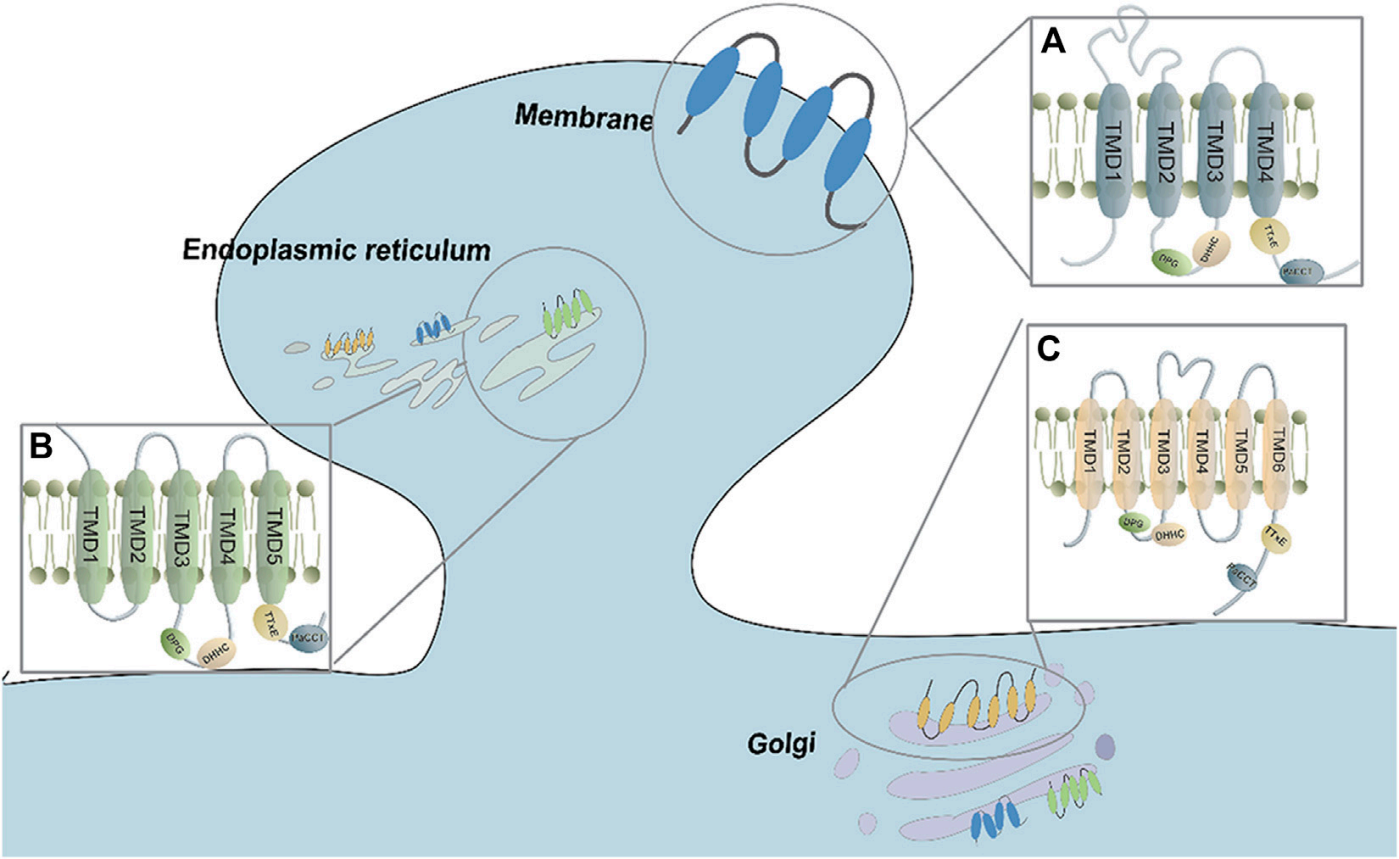
The transmembrane domain and conserved sequences of the ZDHHC family. **(A)** Most ZDHHCs possess 4 TMDs and DHHC-CDR sequences that are located before TMD3. The proteins are mainly distributed in the Golgi, endoplasmic reticulum, and plasma membrane. **(B)** ZDHHC4 and ZDHHC24 have 5 TMDs, and the DHHC-CDR sequence is located before TMD3. They are mainly found in the Golgi and endoplasmic reticulum. **(C)** ZDHHC13, ZDHHC17 and ZDHHC23 have 6 TMDs, and the DHHC-CDR se-quence is located before TMD4. They are mainly found in the Golgi, endoplasmic reticulum, and plasma membrane.

The representative structure of zDHHC proteins includes the TMD (transmembrane domain) and several conserved domains, such as DHHC, DPG (Asp–Pro–Gly), TTxE (Thr-Thr-x-Glu), and Montoro have identified a motif at the C terminus of DHHC enzymes that they have named the palmitoyltransferase conserved C-terminus (PaCCT) motif (González Montoro et al., 2009). Most zDHHC isoforms contain four TMDs (Rana et al., 2019). However, according to topology prediction studies, some zDHHC members may deviate from the traditional four-TMD construction. For example, zDHHC4 and zDHHC24 are predicted to have five TMDs, while zDHHC13, zDHHC17, and zDHHC23 are predicted to have six TMDs (Zaballa and van der Goot, 2018) (Figure 1). The helices of TMDs form a cavity structure that allows fatty acyl chains to be bound. This structure shared among all members of the zDHHC family.

**FIGURE 2:**
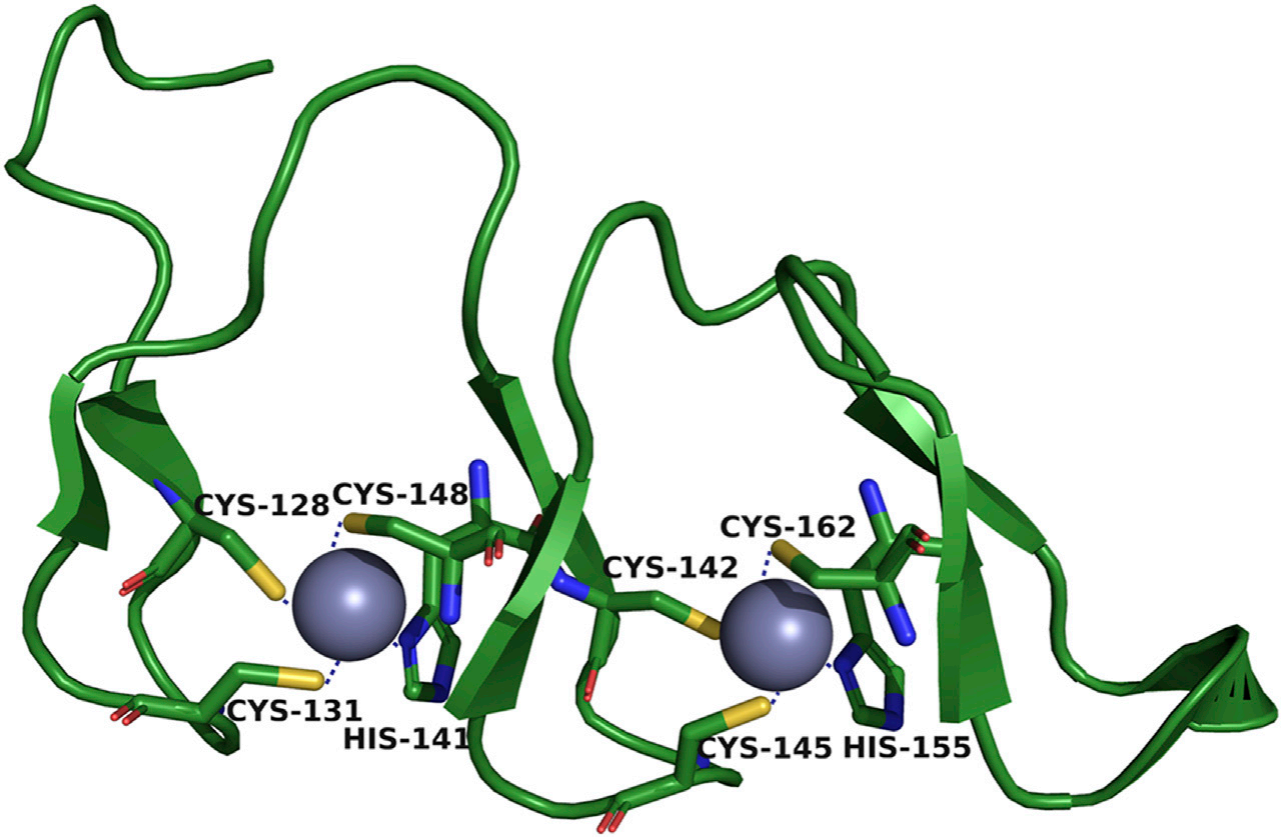
Tetrahedral coordination environment of zDHHC-CRD. One of the purple spheres carries epitaxial Zn^2+^. Three Cys and one His are bound around the spheres.

The DHHC domain is also known as the CRD (cysteine-rich domain) because it contains 50 conserved cysteine and histidine residue fragments. It is an important structure for catalytic reactions (Rana et al., 2019). The zinc finger motifs shared by all zDHHC family members form a tetrahedral coordination environment for Zn^2+^ with three cysteines and one histidine of the CRD (Bonchuk et al., 2022) (Figure 2). This structure constrains the active site of DHHC so that Zn^2+^ is not directly involved in catalytic reactions when located in this space (Reddy et al., 2017). However, the lack of direct involvement does not mean that Zn^2+^ is dispensable for zDHHC protein-mediated S-acylation. By analyzing the activity of zDHHC3, the binding of Zn^2+^ to cysteine residues in the CRD was determined to be needed for the structural integrity of zDHHC proteins (Gottlieb et al., 2015). Although the CRD is an important structural domain for catalytic reactions, several studies suggest that the CRD is not involved in the specific recognition of protein substrates (Malgapo and Linder, 2021). The role that the CRD plays in substrate specificity remains unclear, and future in-depth studies could be carried out on CRD-substrate complexes.

Both the C- and N-terminal ends of zDHHC proteins are located in the inner cell membrane as C- and N-terminal cytoplasmic tails. These cytoplasmic tails are the regions with the greatest sequence diversity within the zDHHC protein and mediate protein‒protein interactions, particularly facilitating substrate interactions and thus the acyl transfer process (Jiang et al., 2018). Although the C-terminal structural domain is more variable in zDHHC enzymes than the CRD, this region also contains the TTxE motif and the PaCCT motif, two structural domains that are to some extent conserved throughout the zDHHC family. TTxE is a motif located in the short helix segment (αʹ1) after TMD4, where x is any amino acid residue. The Thr241 of TTxE binds to the Asp of CRD with hydrogen bonds, and it was shown that the substitution of TTxE by the Ala-Ala-X-Glu (AAxE) mutant significantly reduced enzymatic activity (Rana et al., 2018a). In contrast, two mutated forms (I182S/L184V or M189L/V190C) in relatively conserved sequences outside of the TTxE motifs in zDHHC3 and zDHHC7 did not affect zDHHC enzymatic activity (Greaves et al., 2017). Thus, the exact chemical effect of this contact between TTxE and CDR on catalysis is not yet clear. Similarly, the PaCCT motif is conserved in most zDHHC enzymes, with the third and 11th residues being the most conserved among the 16-residue motifs (Zaballa and van der Goot, 2018). The PaCCT of human zDHHC20 has 16 residue motifs (Asn257-Gly272), among which Asn266 highly conserved interacts with the hydrogen bonds of the Leu261 backbone and the Ser260 side chain, and Asn266 may play an important role in the structural integrity of the enzyme (Stix et al., 2020).

## 3 The catalytic mechanism of zDHHCs

**FIGURE 3:**
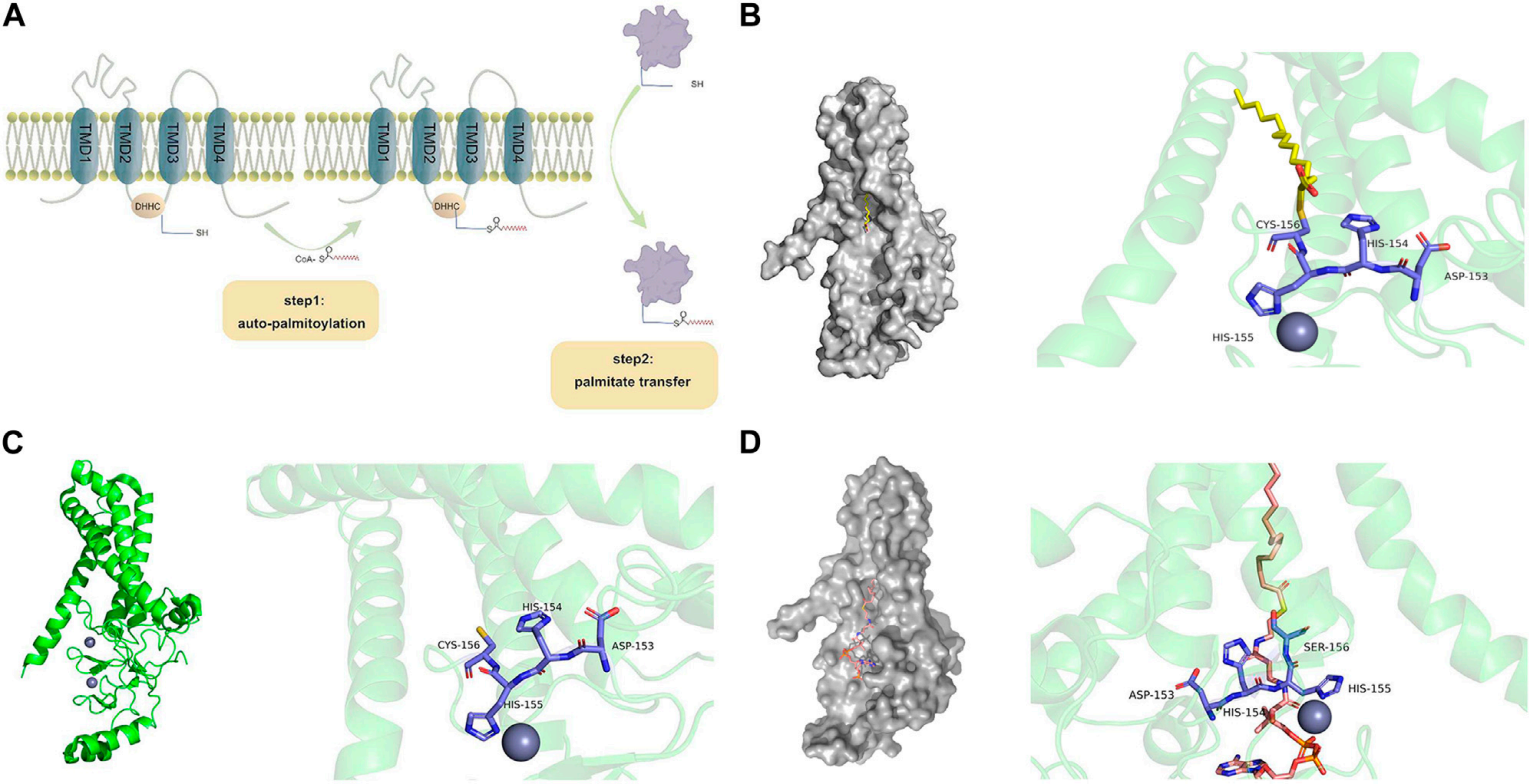
Structural illustrations of zDHHC-mediated protein palmitoylation. **(A)** Two-step catalytic mechanism of zDHHC proteins. CoA binds to the cysteines of long-chain fatty acids to form fatty acyl-CoA. The first step of fatty acyl-CoA facilitates the autoacylation of zDHHC at the membrane, which causes fatty acid lipids to bind to sulfhydryl groups in the cysteine-rich region of zDHHC. The second step brings the cysteine-carrying substrate close to the cell membrane, ultimately binding the fatty acid to the substrate protein. **(B)** Structure of human zDHHC20 palmitoyltransferase, irreversibly inhibited by 2-bromopalmitate. The surface structure of zDHHC20 is gray (PDB entry 6BML (Rana et al., 2018b)), 2-bromopalmitate is colored by element (C-yellow, H-gray, O-red), and the side chains involved in the zDHHC20-CRD catalytic triplex are indicated by small rods and colored by element (C-purple, H-gray, N-blue, O-red, S-yellow). 2-Bromopalmitate irreversibly binds with zDHHC20 and promotes the alkylation of cysteines on DHHC motifs to inhibit autopalmitoylation. **(C)** The structure of human zDHHC20 (PDB entry 6BML (Rana et al., 2018b)) is indicated in PyMOL. TMD 1-4 is indicated as a green helix. The two zinc ions are represented as grayish purple spheres. Side chains involved in the zDHHC20-CRD catalytic triplex are represented by small rods and colored by element (C-purple, H-gray, N-blue, O-red, S-yellow). **(D)** The crystal structure of human zDHHS20 bound to palmitoyl coenzyme A [PDB entry 7KHM (Lee et al., 2022)] is indicated by PyMOL. The zDHHC surface structure is gray, palmitoyl coenzyme A is colored by element (C-orange, H-gray, N-blue, O-red, S-yellow), and the fatty acyl chain is inserted into a hydrophobic pocket within the transmembrane region of the protein. The zDHHC20-CRD catalytic triplex involves a side chain indicated by a small bar that is colored by element (C-purple, H-gray, N-blue, O-red, S-yellow). zDHHC binds to palmitoyl coenzyme A, and the cysteine at position 156 becomes a serine.

Generally, PATs palmitoylate their substrates using a two-step catalytic mechanism, often referred to as the “ping-pong” mechanism (Rana et al., 2018a) (Figure 3A). In the first step, autopalmitoylation ccurs in the zDHHC cysteine side chain, which triggers the rapid transfer of palmitic acid to this site and the formation of a palmitoyl-enzyme intermediate. In the second step, this intermediate binds to the substrate, resulting in a slower reaction step in which the acyl chain is transferred from the zDHHC protein to the Cys on the substrate protein. Eventually, zDHHC is regenerated and ready for the next reaction (Rana et al., 2018a).

### 3.1 Autopalmitoylation of zDHHC proteins

By mutating the cysteine side chain of zDHHC proteins and performing mass spectrometry on some zDHHCs, the autopalmitoyl site was found to be located on the cysteine side chain of DHHC (Collins et al., 2017). A classical inhibitor of palmitoylation, 2-BP (2-bromopalmitate), suppresses autopalmitoylation by promoting the alkylation of cysteines in the DHHC region, further confirming that DHHC the a key site for catalysis reactions (Rana et al., 2018a) (Figure 3B). The zDHHC protein crystal structure was first discovered in human zDHHC20 and zebrafish zDHHC15, revealing the structural basis of autopalmitoylation (Lan et al., 2021). The active site in zDHHC20 is composed of Asp153, His154, and Cys156, which form a linear catalytic triad-like arrangement (Figure 3C). Asp153 polarizes His154, which acts as a universal base and deprotonates Cys156 into a thiolate during palmitoylation. The activated cysteine then acts as a nucleophile, attacking the fatty acyl-CoA carbonyl carbon. After the above series of reactions Cys156 of the zDHHC20 active site is changed to Ser156 (Figure 3D), and the zDHHC-palmitate intermediate is prepared for the next step (Malgapo and Linder, 2021).

### 3.2 zDHHC protein substrate recognition

Currently, the specific recognition of substrates by zDHHC proteins can be explained in two ways. First, all members of the zDHHC family have specific sequences at their N- or C-terminus that correlate with substrate recognition. For instance, zDHHC13 and 17 have anchor protein repeats near their N-termini that are conjugated to Huntington proteins in neurons (Philippe and Jenkins, 2019), while zDHHC5 and zDHHC8 recognize specific substrates through their C-termini PDZ domain (Thomas et al., 2012). Second, the specific structure and amino acid sequence of the substrate can affect their recognition by zDHHCs. For example, amino acids 93–111 in SNAP25 are a flexible molecular spacer that ensures efficient coupling of SNAP25 to the zDHHC17 interaction and S-acylation of SNAP25 (Salaun et al., 2020). Finally, there is still a lack of important evidence linking specific proteins to specific zDHHCs, which should be explored in the future.

## 4 Role of zDHHCs in neurobiology

Protein palmitoylation regulates the differentiation of neural progenitors into neurons and promotes axonal and dendritic growth, which is essential for neuronal development (Koster and Yoshii, 2019). In mature neurons, palmitoylation dynamically regulates protein localization and transport between the synaptic membrane and intracellular compartments, such as the Golgi apparatus and ER (endoplasmic reticulum), during synaptic plasticity (Buszka et al., 2023). As the key enzyme of palmitoylation, the function of zDHHCs is closely related to its distribution within neurons, which in turn affects synapse function.

### 4.1 zDHHC in neuronal cytosol

Neuronal development and plasticity are inextricably linked to zDHHC regulation (Kerkenberg et al., 2021b). zDHHC is located in multiple membrane regions associated with the ER, Golgi apparatus, and plasma membrane (PM) (Globa and Bamji, 2017). Only a few zDHHCs, such as zDHHC5, zDHHC8, and zDHHC14, are highly distributed in the PM. Early biochemical and localization studies suggested that most zDHHC enzymes reside in the ER and Golgi apparatus (He et al., 2014). With more research, it was found that most Golgi-localized zDHHCs are concentrated in the cis-Golgi (Ernst et al., 2018). Since zDHHC plays an important role in the nervous system, the subcellular localization of zDHHC in neurons and the related functions have also become research focuses.

#### 4.1.1 zDHHCs and the Golgi apparatus

The Golgi plays a sorting role in the secretory pathway, and the Golgi is where most palmitoylated substrates are first palmitoylated and then transported to other subcellular organelles, such as the PM (Rocks et al., 2010). A variety of zDHHCs play important roles in the Golgi. The zDHHC17 enzyme present in the Golgi can palmitoylate the protein kinase TrpM7 and regulate embryonic development and the pathogenesis of several common diseases (Gao et al., 2022). The Golgi-localized palmitoyltransferase zDHHC21 has been identified as a key enzyme controlling an acylation-dependent dual ciliary targeting pathway (Kumeta et al., 2018). There are dramatic differences in the substrate affinity and S-acyltransferase activity of zDHHC enzymes. zDHHC17 and zDHHC13 are efficient at protein binding, and zDHHC 3 and zDHHC 7 are efficient at protein S-acylation (Lemonidis et al., 2014). zDHHC17 and zDHHC13 exhibit very strong substrate specificity (Lemonidis et al., 2017). zDHHC17 and zDHHC13 in the Golgi interact with CALCOO1 and participate in the phagocytosis of advanced bodies (Nthiga et al., 2021). zDHHC3 and zDHHC7 have different specificities for different substrates, mainly related to their localization in the cis-Golgi and trans-Golgi regions (Kilpatrick et al., 2016). The role of Golgi-residing zDHHCs in neuronal cells has also been demonstrated. Among zDHHCs, zDHHC3 is stably localized in the Golgi apparatus (Niki et al., 2023). JAK1 palmitoylation is important for neuronal cell medium-dependent signaling and neuronal survival, and zDHHC3 or zDHHC7 can bind to palmitoylated JAK1 (Hernandez et al., 2023). zDHHC7 affects anxiety-related behaviors only in females, a finding that may be used to understand the differences in hormone receptors, neuronal morphology, synaptic plasticity, and molecular signaling pathways between males and females (Kerkenberg et al., 2021a). Disruption of the interaction between SNAP25 and zDHHC17 leads to the loss of stable membrane binding of this protein in neuroendocrine cells (Greaves et al., 2022).

#### 4.1.2 zDHHCs and the ER

The ER is the site of the production of all transmembrane proteins and lipids, including those localized in the ER itself, the Golgi apparatus, lysosomes, intracellular vesicles, the PM, and extracellular compartments. Proteins in the secretory pathway contain many disulfide bonds and functional cysteines at the S-palmitoylation site (Bechtel et al., 2020). There are also proteins in the ER that can be dynamically regulated by palmitoylation (Harada et al., 2020). Dynamic coupling between mitochondria and the ER forms a new subcellular structure, the MAM (mitochondria-associated endoplasmic reticulum membrane), which is strongly implicated in NDs (neurodegenerative disorders) (He et al., 2023). The ER can be coupled to mitochondria in addition to being tethered to the PM via junctophilins (JPH1-JPH4). It is essential for Ca regulation to trigger neuronal excitability as well as Ca homeostasis in nonexcitable cells. Palmitoylation may also enhance the tethering of the ER to the PM by Junctophilins (Jiang et al., 2019). SelK (Selenoprotein K) accelerates the transport of the palmitoylated fatty acid transporter enzyme CD36 from the ER to the Golgi, thereby promoting the PM distribution of CD36 *in vivo* and *in vitro* (You et al., 2022). zDHHC6 present on the ER membrane forms a SelK/zDHHC6 complex with SelK, an ER transmembrane protein that has been shown to be important for ER stress and calcium-dependent signaling, which catalyzes the transfer of fatty acids, such as palmitic acid, to the cysteine residues of target proteins to initiate palmitoylation. This process may be involved in the molecular mechanisms that modulate immunity and cancer (Marciel and Hoffmann, 2019).

### 4.2 zDHHCs in neuronal dendrites

zDHHCs affect dendrite growth and branching. Synaptic activity upregulates the expression of miR-134 (microRNA-134), which regulates dendrite growth and spine formation. miR-134 directly interacts with the mRNA encoding the palymitoyl transferase zDHHC9. miR-134 suppresses the expression of targets such as zDHHC9 and thus the membrane localization of key signaling molecules (Chai et al., 2013). zDHHC9 deficiency in hippocampal cultures results in shorter dendritic spindles and fewer inhibitory synapses, and zDHHC9 promotes dendritic growth by palmitoylating the GTPase Ras and inhibiting synapse formation by palmitoylating the other GTPase TC10 (Shimell et al., 2019). Knockdown of zDHHC15 in primary rat hippocampal cultures reduces dendritic growth and branching. In addition, knockdown of zDHHC15 reduces palmitoylation of PSD-95 and its trafficking to dendrites, resulting in an overall decrease in the density of excitatory synapses formed on mutant cells (Shah et al., 2019). zDHHCs also play a role in dendritic pruning and refinement. zDHHC15 is needed for Sema3F/Nrp2 (secreted semaphorin 3F/neuropilin-2)-dependent dendritic spine pruning and is a Sema3A/Nrp1 (semaphorin 3A/neuropilin-1)-dependent substrate. Dendritic refinement is a necessary process (Koropouli et al., 2023). zDHHCs also affect related dendritic proteins. zDHHC17 palmitoylation is essential for the localization and function of ankyrin-B in dendrites (Gupta and Jenkins, 2023). zDHHC8 increases the stability of AnkG-190 (190 kDa isoform) dendrites (Piguel et al., 2023). Neuronal activity also mediates the regulation of dendritic palmitoylation by zDHHCs. With altered neuronal activity, zDHHC5 is internalized from the synaptic membrane to the dendritic shaft and contributes to dendritic shaft δ-catenin palmitoylation (Brigidi et al., 2015).

#### 4.2.1 zDHHCs and glutamate receptors

In the vertebrate central nervous system, GluR (glutamate receptors) play a central role in excitatory synaptic transmission and plasticity in the brain and are critical for memory formation, learning, emotion and behavior (Sohn and Park, 2019). GluRs are composed of ionotropic and metabotropic GluRs (mGluRs and iGluRs). Among them, iGluRs mainly include AMPARs (α-amino-3-hydroxy-5-methyl-4-isoxazolepropionate-type receptors) and NMDARs (N-methyl-D-aspartate-type receptors), which are mainly responsible for glutamate-mediated postsynaptic excitation of neurons (Mori and Mishina, 2003).

Palmitoylation regulates the synaptic expression, intracellular localization, and membrane trafficking of iGluRs in neurons, and the dysregulation of iGluR palmitoylation can lead to brain disorders such as seizures (Hayashi, 2021). AKAP150 (A Kinase-anchored protein 150) organizes kinases and phosphatases to regulate AMPARs, which are critical for synaptic plasticity. Palmitoylation of AKAP150 controls its subcellular localization to maintain palmitoylation of AKAP150 to maintain proper basal and activity-dependent regulation of synaptic AMPAR subunit composition (Purkey et al., 2018). Upregulated expression of ZDHHC2 contributes to the upregulation of AKAP150 palmitoylation and accumulation of the AMPAR GluA1 subunit in inflammation model mice, demonstrating a potential therapeutic strategy for persistent pain and inflammation (Li et al., 2021). NMDARs play a key role in synaptic signaling in HD. In the YAC128 mouse model, enhanced activity of extrasynaptic 2B-NMDAR (GluN2B-type NMDAR) in striatal neurons leads to increased activation of the cell death pathway, which predisposes the striatum to degeneration. Altered regulation of GluN2B palmitoylation levels by zDHHC17 may contribute to the cell death signaling pathway in HD (Kang et al., 2019).

zDHHC3 can modulate the synaptic clustering of glutamate receptors (Shah et al., 2023). Moreover, overexpression of zDHHC3 leads to the overpalmitoylation of the AMPAR subunit GluA1, preventing its activity-dependent migration to the PM. As a result, the AMPAR current amplitude decreases, inhibiting synaptic plasticity and hippocampus-dependent memory and, therefore, affecting learning and memory (Spinelli et al., 2017). Abnormal GluA1 palmitoylation of synaptic AMPARs leads to hyperexcitability and thus to seizures (Itoh et al., 2018). zDHHC regulation of AMPARs GluA1 must be at an appropriate scale when treating pain to ensure that it does not affect learning and memory or induce seizures. It was also found that zDHHC3 is a target of miR-134-3p. miR-134-3p is involved in aluminum neurotoxicity by targeting the zDHHC3-AMPARs axis, which could serve as a potential biomarker or useful target (Song et al., 2023).

In addition to its role in iGluRs, zDHHCs play an important role in the regulation of mGluRs. Palmitoylation of Erα (estrogen receptor α) and Erβ (estrogen receptor β) in the nervous system is needed for membrane surface localization and the mediation of downstream signaling via mGluR activation (Meitzen et al., 2019). zDHHC7 and zDHHC21 have been implicated in this signaling mechanism (Tonn Eisinger et al., 2018).

#### 4.2.2 zDHHCs and scaffold proteins

Scaffold proteins regulate coordinated neurotransmission by immobilizing and aggregating receptors and adhesion molecules. zDHHCs can mediate a variety of scaffolding proteins, which in turn affects neural signaling. Gephyrin is a core scaffolding protein of many inhibitory synapses that aggregates glycine as well as a major subgroup of GABA receptors, GABAARs (GABA type A receptors). Palmitoylation of gephyrin by zDHHC12 contributes to the dynamic and functional regulation of GABAergic synapses (Dejanovic et al., 2014). Ankyrin-B is an intracellular scaffolding protein that is localized in dendrites and axons in mature neurons, and zDHHC17 is a key mediator of ankyrin-B palmitoylation in heterologous cells and neurons (Gupta and Jenkins, 2023). Early study uncovered a critical role for PSD-95 (protein postsynaptic density protein 95) palmitoylation in regulation of synaptic AMPARs content (El-Husseini Ael et al., 2002). Increased PSD-95 palmitoylation is associated with increased levels of surface AMPARs (Jeyifous et al., 2016). Dynamic modulation of palmitoylation of PSD-95 can modulate AMPARs (Chowdhury and Hell, 2019). The scaffolding PSD-95, whose palmitoylation is mediated by zDHHC-2, -3, -7, and -15, regulates the integration and stability of AMPAR in excitatory postsynaptic membranes (Shah et al., 2019; Liu et al., 2022). zDHHC2 moves to the postsynaptic density in an activity-sensitive manner and mediates the palmitoylation of PSD-95. This process increases the accumulation of synaptic PSD-95 and AMPAR in response to activity blockade, which promotes homeostatic plasticity (Fukata and Fukata, 2010). The S-acyltransferase zDHHC2 mediates the dynamic S-acylation of PSD-95 and AKAP79/150, which also affects the synaptic targeting of AMPA receptors (Salaun et al., 2017). Moreover, IL-6 upregulates the expression of zDHHC2 and zDHHC3, thereby promoting the palmitoylation of PSD-95 (Liu et al., 2022).

### 4.3 zDHHCs in neuronal axons

Proper axonal growth during nervous system development is critical for synaptic transmission and nervous sys-tem function. However, little is known about which PATs are present in neuronal axons. Elucidating which PATs are present in neuronal axons can provide insights into their role in synapses. Thus, in 2020, mammalian DRG (dorsal root ganglion) neurons were utilized to determine the subcellular distribution of PATs, and only two PATs, zDHHC5 and zDHHC8, were found to be present in axons (Collura et al., 2020). Palmitoylation of proteins regulating axon growth and branching in zDHHC8-deficient mice results in reduced axon growth and terminal branching, and reintroduction of the active zDHHC8 protein prevents these changes (Mukai et al., 2015). In addition to the two types of zDHHC that have been demonstrated in axons, there are other axon-associated zDHHCs. After ONC (optic nerve crush), the cell bodies and distal axons of most RGCs (retinal ganglion cells) are injured. However, zDHHC17 ensures the integrity of distal axons in healthy optic nerves (Niu et al., 2020). zDHHC14 colocalizes with its substrate PSD93 at axon initiating segments and promotes its function of organizing ion channels at this site (Sanders et al., 2020). zDHHC17 is needed for normal axon growth *in vivo* and *in vitro* (Shi et al., 2015). Axons are critical for synaptic transmission and nervous system function, and some zDHHCs are localized in axons, affecting nervous system development and function. For example, palmitoylation of δ-catenin is critical for synaptic plasticity and memory formation. zDHHC3-mediated ð-catenin palmitoylation also plays an important role in the development of neuropathic pain. Blocking this mechanism may be therapeutic for patients with neuropathic pain (Zhang et al., 2018).

## 5 The role of zDHHC in neurological diseases

Protein palmitoylation plays an important role in the regulation of physiological functions in the brain. zDHHC enzymes involved in palmitoylation has been shown to be regulated by posttranscriptional modifications in terms of stability, localization, and function. Moreover, zDHHcs have been found to play a role in neuronal morphogenesis, thereby regulating neurologically related diseases (Zmuda and Chamberlain, 2020). Each zDHHC has a different substrate (Cho and Park, 2016; Globa and Bamji, 2017). Many studies have reported that different zDHHCs are present in different brain regions (Cembrowski et al., 2016). They are closely associated with neurological disorders such as (AD) Alzheimer’s disease, HD, SCZ, (XLID) X-linked intellectual disability and (ADHD) attention deficit hyperactivity disorder. Table 1 shows the associations between zDHHC proteins and human neurological disorders. In the future, these proteins can play an important role in the diagnosis and management of related disorders.

**TABLE 1:** Human neurological disorders and zDHHCs.

Disorder	zDHHC isoforms	Disease related substrate	Specific substrate	Subcellular localizations	Major expression patterns in the brain
Alzheimer’s disease	zDHHC7	APP	PSD-95, GAP43, SNAP25, SNAP23, Gas, Gaq, Gai2, CSP, GABAAg2, eNOS, STREX, Fyn, BACE1, NDE1, NDEL1, NCAM140, sortillin, DE10A2, CSP, GABAAg2, eNOS, NCAM, Neurochondrin, APP, Stathmin 2/SCG10, PPT1	Golgi	cortex, olfactory bulb, CA1 hippocampus
	zDHHC12	APP Gephyrin	ABCA1, APP, Gephyrin	Golgi, ER	
	zDHHC15	Fyn APP	PSD-95, GAP43, SNAP25b, CSP, GABAAg2, Fyn, BACE1, CD151, CIMPR, sortillin, APP, DAT, GP130, DAT	Golgi	CA1 hippocampus
	zDHHC21	Fyn APP	Fyn, eNOS, Lck, ABCA1, APP, Gαi2, PI4KIIα, 5-HT1AR	Golgi	CA1 hippocampus
Huntington’s disease	zDHHC13	Huntingtin	Huntingtin, GAD65, SNAP-25	Golgi, ER	CA1 hippocampus
	zDHHC17	Huntingtin SPRED1 SPRED3	Lck, SNAP25, SNAP23, CSP, Huntingtin, GluA1/2, GAD65, STREX, PSD-95, synaptotagmin I, ClipR59, MPP1/p55, JNK, SPRED1, SPRED3, DAT, TrpM7	Golgi	Ubiquitous
X-linked intellectual disability	zDHHC9	N-Ras, H-Ras TC10	Cofactor GCP16, needed for enzymatic activity, STREX, H-Ras, N-Ras, TC10	Golgi, ER	Ubiquitous
	zDHHC15		PSD-95, GAP43, SNAP25b, CSP, GABAAg2, Fyn, BACE1, CD151, CIMPR, sortillin, APP, DAT, GP130, DAT	Golgi	CA1 hippocampus
Schizophrenia	zDHHC2		PSD-95, SNAP25, SNAP23, eNOS, Fyn, NDE1, NDEL1, CD151, CKAP4, ABCA1, GAP43, Tetraspanins, CD9/CD151, CKAP4/p63, DAT	Golgi, ER	cortex, CA1 hippocampus
	zDHHC5		STREX, flotillin-2, GRIP1, d-catenin, somatostatin receptor 5, δ-catenin. EZH2	Membrane	Ubiquitous
	zDHHC8	PSD-95 Akt	eNOS, SNAP25, paralemmin-1, GAD65, PSD-95, PSD93, ABCA1, PICK1, GRIP1, DAT, Akt	Golgi	cortex, olfactory bulb, hippocampus
	zDHHC18	CDC42	H-Ras, N-Ras; H-Ras, Lck, CDC42	Golgi	
Attention deficit hyperactivity disorder	zDHHC2	DAT	PSD-95, SNAP25, SNAP23, eNOS, Fyn, NDE1, NDEL1, CD151, CKAP4, ABCA1, GAP43, Tetraspanins, CD9/CD151, CKAP4/p63, DAT	Golgi	cortex, CA1 hippocampus
	zDHHC3	DAT	PSD-95, SNAP25, SNAP23, Gas, Gaq, Gai2, CSP, GABAAg2, eNOS, GluA1/2, GAD65, STREX, Fyn, BACE1, NDE1, NDEL1, NCAM140f, CaMKIg, NR2A/B, Neurochondrin, DAT, PPT1	Golgi	cortex, olfactory bulb, hippocampus
	zDHHC8	DAT	eNOS, SNAP25, paralemmin-1, GAD65, PSD-95, PSD93, ABCA1, PICK1, GRIP1, DAT, Akt	Golgi	CA1 hippocampus
	zDHHC15	DAT	PSD-95, GAP43, SNAP25b, CSP, GABAAg2, Fyn, BACE1, CD151, CIMPR, sortillin, APP, DAT, GP130, DAT	Golgi	Ubiquitous
	zDHHC17	DAT	Lck, SNAP25, SNAP23, CSP, Huntingtin, GluA1/2, GAD65, STREX, PSD-95, synaptotagmin I, ClipR59, MPP1/p55, JNK, SPRED1, SPRED3, DAT, TrpM7	Golgi	
Glioma	zDHHC5	EZH2	STREX, flotillin-2, GRIP1, d-catenin, somatostatin receptor 5, δ-catenin. EZH2	Plasma membrane	Ubiquitous
	zDHHC15	GP130	PSD-95, GAP43, SNAP25b, CSP, GABAAg2, Fyn, BACE1, CD151, CIMPR, sortillin, APP, DAT, GP130, DAT	Golgi	CA1 hippocampus
	zDHHC19	Smad3	R-Ras, Smad3	Plasma membrane	
	zDHHC16	SEDT2	SEDT2	ER	
Neuronal Ceroid Lipofuscinosis	zDHHC3	PPT1	PSD-95, SNAP25, SNAP23, Gas, Gaq, Gai2, CSP, GABAAg2, eNOS, GluA1/2, GAD65, STREX, Fyn, BACE1, NDE1, NDEL1, NCAM140f, CaMKIg, NR2A/B, Neurochondrin, DAT, PPT1	Golgi	
	zDHHC7	PPT1	PSD-95, GAP43, SNAP25, SNAP23, Gas, Gaq, Gai2, CSP, GABAAg2, eNOS, STREX, Fyn, BACE1, NDE1, NDEL1, NCAM140, sortillin, DE10A2, CSP, GABAAg2, eNOS, NCAM, Neurochondrin, APP, Stathmin 2/SCG10, PPT1	Golgi	cortex, olfactory bulb, CA1 hippocampus
Major depressive disorder	zDHHC21	5-HT1AR	Fyn, eNOS, Lck, ABCA1, APP, Gαi2, PI4KIIα, 5-HT1AR	Golgi	CA1 hippocampus
Epilepsy	zDHHC8	AMPA	eNOS, SNAP25, paralemmin-1, GAD65, PSD-95, PSD93, ABCA1, PICK1, GRIP1, AMPA,DAT	Golgi	cortex, olfactory bulb, hippocampus

### 5.1 zDHHCs in AD

AD is one of the most common neurodegenerative diseases, manifested by cognitive decline and progressive memory loss and characterized by neuronal dysfunction. There are two main neuropathological features of AD associated with palmitoylation, the most widely known of which is neurotoxic extracellular Aβ (β-amyloid) aggregation forming amyloid plaques (Zhang et al., 2019), and the other is intracellular NFTs (neurofibrillary tangles) (Lopes et al., 2022).

Aβ aggregation to form amyloid plaques is mainly closely related to APP (amyloid precursor protein) and the APP hydrolysis-related enzymes β-secretase (β-site APP cleaving enzyme 1, BACE1) and γ-secretase (Wang et al., 2021). APP, BACE1, and γ-secretase can all be palmitoylated and modified or regulated by palmitoylated proteins. Among them, Cys 186 and Cys 187 of APP can increase the interaction of APP with lipid rafts through palmitoylation, which enhances the gene processing of amyloid proteins and leads to increased γ-secretase-mediated cleavage (Shafi et al., 2021). Palmitoylation of BACE1 occurs at the transmembrane and C-terminal cytoplasmic structural domains of four cysteine residues (Cys474, Cys478, Cys482 and Cys485) (Shah, 2020). It was demonstrated that S-palmitoylation of BACE1 reduces Aβ deposition and thus attenuates memory impairment in AD model mice (Andrew et al., 2017). It was also clarified that palmitoylation of BACE1 in the cytoplasmic tail Cys residues affects AD (Wen et al., 2022). By using a mouse model of AD, it was confirmed that the reduced levels of insoluble Aβ and amyloid deposition in the frontal cortex were associated with a palmitoylated defective form of γ-secretase (Bhattacharyya et al., 2013).

Studies on APP palmitoylation by PATs are relatively comprehensive. Accumulating evidence has shown that members of the zDHHC family regulate APP in two main ways, thereby influencing the pathogenesis of AD. The first is the direct linkage of zDHHC12 to APP. The binding of zDHHC12 to APP was verified by immunoprecipitation, and as a result, zDHHC12 is also called APP-interacting DHHC protein (AID) (Mizumaru et al., 2009). zDHHC12 (or AID) inhibits APP metabolism and Aβ production by suppressing APP transport. Nonamyloidogenic α-cleavage of APP was dependent on zDHHC12/AID-mediated palmitoylation. Moreover, zDHHC7 and zDHHC21 are involved in the palmitoylation of APP. It has been shown that overexpression of zDHHC7 and zDHHC21 increases the palmitoylation of APP in lipid rafts, which results in APP being more susceptible to cleavage by BACE1 and increased Aβ production (Bhattacharyya et al., 2013).

In addition to the production of amyloid plaques through Aβ aggregation, another neuropathological feature of AD is the formation of intracellular NFTs due to hyperphosphorylated tau, which is caused by Fyn kinase (Lopes et al., 2022). zDHHC21 is the PAT of Fyn (Mill et al., 2009). Recent studies that investigated familial Alzheimer’s disease (FAD) using exome sequencing have revealed the gene variant ZDHHC21 p.T209S. Mutating ZDHHC21 significantly enhances Fyn palmitoylation, contributing to the overactivation of GluN2B-containing NMDARs, which further leads to synaptic dysfunction and neuronal loss. Moreover, the palmitoylation of APP was also increased, resulting in the production of Aβ (Li et al., 2023).

### 5.2 zDHHCs in HD

HD is an autosomal dominant neurodegenerative disease caused by a mutation in the HTT (huntingtin) gene. The mutation is caused when a DNA segment known as a CAG trinucleotide repeat in the huntingtin gene is repeated more than 35 times (Group, 1993). HD patients present cognitive, motor, and psychiatric symptoms (Sun et al., 2017). Palmitoylation is strongly associated with HD, and it has been determined that, compared to normal HTT, mutant HTT has reduced levels of palmitoylation and increased huntingtin-induced toxicity (Lemarie et al., 2021). However, the effect of palmitoylation on HTT function and the role of HTT palmitoylation deficiency in the pathogenesis of HD remain unclear.

First, a yeast two-hybrid screen indicated that zDHHC17 interacts with HTT (Kalchman et al., 1996). Then, the Akr structure located in the N-terminal region of zDHHC13 and zDHHC17 was found to interact with HTT and regulate its transport when fused to the N-terminus of zDHHC3 (al, 2002). zDHHC17 and zDHHC13 (HIP14 and HIP14L) then induce palmitoylation at HTT cysteine 214 (C214) (Huang et al., 2004). Subsequently, in immortalized cell lines and primary neurons carrying the palmitoylation-resistant mutation HTT (C214 to serine, C214S) and in a YAC128 mouse model (full-length human HTT transgenic mice with 128 CAG repeats), it was demonstrated that zDHHC17 and zDHHC13-associated palmitoylation are both decreased in HD, increasing susceptibility to excitotoxicity (Singaraja et al., 2002). It was then further clarified that the autopalmitoylation of zDHHC17 and zDHHC13 and the palmitoylation of many synaptic substrates were also decreased in the YAC128 mouse model (Huang et al., 2011). In the presence of HTT mutations, the interaction of zDHHC17 and zDHHC13 with HTT is altered, resulting in reduced palmitoylation, and it is hypothesized that HD is a disease caused by altered palmitoylation (Sanders and Hayden, 2015). SPRED1 and SPRED3 have been identified as novel substrates of zDHHC17 palmitoylated proteins and may be important in the pathogenesis of zDHHC17-altered palmitoylated HD (Cho and Park, 2016). The relationship between palmitoylation and HD is unclear, and it is currently hypothesized that altered regulation of GluN2B palmitoylation by zDHHC13 may promote the apoptosis of medium spiny neurons, which leads to the de-velopment of HD (Kang et al., 2019)。

Targeting the palmitoylation of HTT, which is dysregulated in HD mutations, to promote neuronal survival has received attention as a potential clinical treatment for the disease. It has been demonstrated that lowering mHTT levels in the brains of HD mice does not rescue abnormal palmitoylation. However, it is possible to normalize the palmitoylation of mHTT in HD patient cells using acyl protein thioesterase (APT). It is also possible to reduce mHTT aggregation and cytotoxicity *in vitro* by promoting palmitoylation (Lemarie et al., 2021). Restoration of palmitoylation levels using the APT1 inhibitor ML348 ameliorated neuropathology, movement disorders, and anxiety-depressive behaviors. Modulation of palmitoylation is important for the treatment of HD (Virlogeux, 2021). In addition to focusing on HTT palmitoylation, a potential risk factor controlling the S-palmitoylation of TRPC5 channels could modulate TRPC5 channel expression and activity, providing new insights into novel therapeutic strategies for HD (Hong et al., 2020).

### 5.3 zDHHCs in SCZ

SCZ is a psychiatric disorder with symptoms including apathy and withdrawal as well as cognitive impairment. It has been reported that protein palmitoylation in the dorsolateral prefrontal cortex of SCZ patients is lower than that in normal subjects (Pinner et al., 2020). The relationship between SCZ and zDHHC family proteins has received extensive attention from researchers in recent years.

The zDHHC8 gene, located in the microdeletion region of chromosome 22q11, was found to be associated with SCZ risk (Woodin, 2001). The current study suggests that zDHHC8 may influence cortical volume and exhibit a positive effect on SCZ (Ota et al., 2013). Interestingly, the correlation of zDHHC8 with SCZ was inconsistent in different populations. A significant association between zDHHC8 and SCZ has been demonstrated in populations from the United States, South Africa, China, and Korea (Shin et al., 2010). However, studies in Japanese and European populations have shown no association between zDHHC8 and SCZ (Saito et al., 2005). Decreased dendritic spine numbers may be a pathogenic mechanism of schizophrenia. The loss of dendritic spines can lead to numerous manifestations that are associated with schizophrenia, such as poor synaptic strength and dysfunctional connectivity (Mukai et al., 2015). The palmitate cycle on PSD-95 modulates synaptic strength and regulates aspects of activity-dependent plasticity (El-Husseini Ael et al., 2002). zDHHC8-deficient mice have low spin densities during neurodevelopment, which can be relatively normalized by the administration of the active zDHHC8 protein, as PSD-95 is a substrate for zDHHC8, and its palmitoylation regulates dendritic spine density (Mukai et al., 2008). In addition to zDHHC8, analysis of a genome-wide association study and expression quantitative trait loci data from more than 9000 SCZ patients revealed that zDHHC18 was one of the highest-scoring genes associated with SCZ (Zhao et al., 2018). Moreover, researchers refined the variants of zDHHC2 based on its identification as a candidate gene for SCZ and validated that these variants were risk factors for SCZ in a Chinese Han population (Zhang et al., 2020). Recently, the structure of zDHHC5 was found to be altered in SCZ patients (Shimell et al., 2021). The number of DHHC family members that have been found to be associated with SCZ has continuously increased in recent years, but the molecular mechanisms underlying their association with SCZ have been relatively superficially studied and are an area that should be explored in the future.

### 5.4 zDHHCs in XLID

Intellectual disability is characterized by significantly lower intelligence than the general population, as well as impaired adaptive behavior and lack of daily living skills, social skills, and communication skills (Frints et al., 2019).

Specific RT‒PCR analysis of zDHHC15 in lymphocytes from XLID patients showed the presence of zDHHC15 transcript variants in patient samples, and it was hypothesized that deleting the zDHHC15 transcript contributed to the XLID phenotype, suggesting that zDHHC15 is a strong candidate gene for the diagnosis of nonsyndromic XLID (Mansouri et al., 2005). A recent study found that zDHHC15 plays a crucial role in neuronal differentiation in zebrafish (Wang et al., 2015). However, zDHHC15 remains one of the less-studied zDHHCs, and its mechanism of action and relationship with XLID remain unclear.

Compared to zDHHC15, zDHHC9 has been studied thoroughly in the context of XLID. Loss-of-function mutations in zDHHC9 were found in XLID patients. zDHHC9 loss of function in hippocampal cultures leads to shorter dendritic spindles and fewer inhibitory synapses, which alters the ratio of excitatory to inhibitory signals in Zdhhc9-deficient cells (Raymond et al., 2007). zDHHC9 mutation alters the expression level and cellular localization of H-Ras and N-Ras in membrane-localized GTPases (guanosine triphosphate hydrolases), regulates dendritic growth, and promotes inhibitory synapse formation by palmitoylating TC10 (Raymond et al., 2007). Two naturally occurring variants of zDHHC9 reduce the steady-state levels of zDHHC9 self-palmitoylation through different mechanisms, affecting palmitoylation target proteins involved in intellectual development (Mitchell et al., 2014). zDHHC9 mutation inactivates its PAT activity, which is associated with impairment of language and memory in XLID (Kouskou et al., 2018). Activation and stabilization of zDHHC9 requires an accessory protein, GCP16, which stabilizes zDHHC9 by preventing zDHHC9 aggregation through the formation of the zDHHC9-GCP16 protein complex. zDHHC9 mutations associated with XLID lead to reduced protein stability and zDHHC9-GCP16 complex formation capacity (Nguyen et al., 2023). In the future, target proteins associated with XLID can be identified from the substrates of stabilized zDHHC9, which will be important for the future diagnosis and treatment of XLID.

### 5.5 zDHHCs in ADHD

ADHD is a heterogeneous neurodevelopmental disorder with a high degree of heritability characterized by inattention, hyperactivity, and impulsivity, which are also present in high novelty responses (Schlag et al., 2022). Several studies have confirmed that ADHD is associated with an imbalance in DA (dopamine) (Vazquez et al., 2022). Both the DAT (DA transporter) and DA receptors, which are associated with DA homeostasis, have been shown to be palmitoylated (Rastedt et al., 2017).

The D1 dopamine receptor belongs to the class A superfamily of GPCRs (G protein-coupled receptors), and both Cys347 and Cys351 of the D1 receptor are palmitoylated (Zareba-Koziol et al., 2018). Palmitoylation may be involved in directing agonist-dependent internalization of D1 receptors via a selective endocytic pathway (Kong et al., 2011). In addition to DA receptors, DAT has also been shown to undergo palmitoylation (Zeppelin et al., 2021). Cys580 was identified as a major palmitoylation site on DAT (Foster and Vaughan, 2011). By using 2BP to examine the functional ramifications of DAT palmitoylation, it was found that Cys580 palmitoylation enhanced the kinetic capacity of DAT, stabilized the metabolism of DAT, and controlled the long-term total transporter level (Foster and Vaughan, 2011).

Palmitoylation of DAT was enhanced by the expression of PATs, including zDHHC2, zDHHC3, zDHHC8, zDHHC15 or zDHHC17 (Bolland et al., 2019). zDHHC15 knockout mice showed normal spatial learning and working memory abilities but a significant increase in novelty-induced locomotion in an open field. During the habituation phase to the new environment, striatal dopamine levels decreased, but extracellular dopamine levels increased. This finding suggests that ZDHHC15-mediated palmitoylation is a novel regulatory mechanism of dopamine in the striatum (Mejias et al., 2021). zDHHC15b is homologous to human zDHHC15, and its expression is upregulated during DA neuron differentiation, whereas knockdown of zDHHC15b reduces DA neuron differentiation and thereby affects DA neuron fate determination (Wang et al., 2015). Among these PATs, zDHHC15 at human Xq13.3 is not only associated with XLID but also closely related to ADHD (Wang et al., 2015).

### 5.6 zDHHCs in glioma

Glioma is a highly aggressive and highly lethal malignant tumor of the central nervous system (Liu et al., 2021). In particular, glioblastoma, a grade IV glioma, is the most aggressive primary brain tumor, with a recurrence rate of up to 90% and a survival rate of only 15 months after diagnosis (Patro et al., 2022). Abnormal protein palmitoylation of glioma brain tissue compared to normal brain tissue in the central nervous system correlates with the abnormal expression of some zDHHCs in gliomas (Tang et al., 2022a).

*In vivo* and *in vitro* experiments have shown that in gliomas with mutations in human TP53 (tumor protein P53), zDHHC5 mediates enhancer of EZH2 (zeste homolog 2) palmitoylation, leading to an altered phosphorylation state of EZH2 that drives glioma development (Gong et al., 2023). Overexpression or knockdown of zDHHC5 affected cell growth, the cell cycle, self-renewal and invasiveness, and the expression level of zDHHC5 was inversely associated with the overall survival of glioma patients (Chen et al., 2017). Propofol promotes glioma growth through zDHHC5 and EZH2, and tight control of Propofol dosage in the clinic leads to better prognosis for patients after surgical removal of tumors (Fan et al., 2022a). Upregulation of both the mRNA and protein expression of zDHHC9 in gliomas correlates with tumor grade (Zhang et al., 2021). Glioma patients with high expression of zDHHC9 have shorter survival prognosis. Knockdown of zDHHC9 in an athymic nude mouse model bearing glioma enhances the survival time of mice (Zhang et al., 2021).

Some zDHHCs have important implications in the immunotherapy of gliomas. zDHHC12 is also aberrantly expressed in gliomas, and knockdown of zDHHC12 reduces glioma cell survival, while overexpression of zDHHC12 not only promotes glioma cell growth but is also associated with immune cell infiltration (Lu et al., 2022). In gliomas, immune cell infiltration leads to immunotherapy resistance. Therefore, the role of zDHHC12 in gliomas may have therapeutic implications for glioblastoma immunotherapy (Tang et al., 2022b). zDHHC17 is also aberrantly expressed in gliomas, and it has been determined that the zDHHC17/MAPK signaling module is essential for promoting radiotherapy resistance in gliomas. It has been determined that genistein can inhibit the zDHHC17/MAPK signaling module, thus reducing radiotherapy resistance in gliomas (Chen et al., 2020).

Some zDHHCs can also be prognostic biomarkers for glioma patients. zDHHC11 and zDHHC22 are favorable prognostic markers, while upregulated expression of zDHHC4 predicts a poor prognosis for patients (Tang et al., 2022b). Recent studies have confirmed a clear link between high zDHHC15 expression and the malignant glioma phenotype, and zDHHC15 plays a key role in promoting the proliferation and migration of glioma cells by activating the STAT3 signaling pathway. Researchers have also suggested that zDHHC15 may be a new prognostic biomarker for patients with glioma and that targeting zDHHC15 could provide a promising strategy for the treatment of glioma (Liu et al., 2023). Local anesthetics may attenuate zDHHC15 transcription, reduce GP130 palmitoylation levels and membrane localization, and thus inhibit IL-6/STAT3 signaling activation. Therefore, local anesthetics may become an option for the treatment of glioblastoma (Fan et al., 2021b).

Some zDHHCs can precisely target gliomas in specific settings. It is of concern that gliomas have different subgroup compositions with plasticity and substantial heterogeneity, which is one of the reasons why genomic analyses have not been successful in guiding the development of precision medicine for these tumors (Nicholson and Fine, 2021). zDHHC18 and zDHHC23 can target different subpopulations of glioma stem cells in specific settings (Chen et al., 2019). zDHHC18 is a mesenchymal glioma stem cell marker, whereas zDHHC23 is a glioma stem cell marker. zDHHC18 is localized in the necrotic zone. In contrast, zDHHC23 is distributed at the border of human glioma tissue (Tang et al., 2022b). zDHHC19-mediated Smad3 palmitoylation promotes the activation of the transforming growth factor-β signaling pathway, and its interaction with EP300 upregulated the expression of mesenchymal markers in the mesenchymal GBM subtype. This study also provides an important therapeutic target for the treatment of gliomas (Fan et al., 2021a). In glioma tissue, not all abnormal zDHHCs were overexpressed compared to those in normal brain tissue, and zDHHC16 was significantly downregulated compared to that in normal brain tissue. The survival time of glioma patients with low expression of zDHHC16 and high expression of epidermal growth factor receptor was shorter than that of patients with high expression of zDHHC16 and low expression of epidermal growth factor. In addition, zDHHC16 can mediate the palmitoylation of SET domain-containing 2 (SEDT2). Elevated palmitoylation of SEDT2 by palmitate B restored zDHHC16 and may be useful for the treatment of patients with glioblastoma due to epidermal growth factor receptor amplification (Fan et al., 2022b).

### 5.7 zDHHCS and other neurological disorders

In addition to the above neurologic disorders, there are other neurologic disorders associated with zDHHC. Mutations in the depalmitoylase gene PPT1 cause infantile NCL (neuronal ceroid lipofuscinosis), an early-onset neurodegenerative disease. zDHHC3 and zDHHC7 induce the palmitoylation of PPT1 *in vivo*, and the palmitoylation of PPT1 leads to a decrease in its activity, which indirectly leads to a subsequent increase in the amount of palmitoylated proteins. This positive feedback loop likely triggers a vicious cycle that exacerbates disease progression (Segal-Salto et al., 2016). Serotonin 1A receptor (5-HT1AR) is associated with MDD (major depressive disorder) and 5-HT1AR palmitoylation is decreased in depressive patients. Moreover, the expression of zDHHC21 is positively correlated with the palmitoylation of 5-HT1AR (Gorinski et al., 2019). Mutations in the zDHHC15 gene are thought to cause several neurodevelopmental disorders, such as hypotonic cerebral palsy, autism, epilepsy, and intellectual disability (Lewis et al., 2021). Recently, zDHHC15 has been recognized as a candidate gene for autism spectrum disorder (ASD). New evidence also suggests that this gene may be associated with other neurodevelopmental disorders (Casellas-Vidal et al., 2023). zDHHC8 may be associated with epileptic seizures in humans, and knockdown of zDHHC8 may have anti-epileptogenic effects on drug-resistant epilepsy. Epilepsy could be treated in the future by modulating AMPA/GluA1-mediated neurotransmission (Yang et al., 2018).

## 6 Future prospects

By studying the role of palmitoylation in neurological disorders, it is known that in neurons, zDHHC binding to the corresponding substrate can modulate neuronal morphology and synaptic plasticity and thus affect signaling in the nervous system. However, the degree of study of zDHHC family substrates is relatively unequal. For example, zDHHC13 and zDHHC17 are relatively well studied, but there are also zDHHC family members, such as zDHHC8, zDHHC9, zDHHC15, and zDHHC18, whose substrates are still unclear. Some enzymes have a wide range of target proteins, while others are highly specific, and there is a lack of detail concerning enzyme-substrate-specific recognition, which has hampered the understanding of the role of palmitoylation in the regulation of neurological functions. This role can be explored by sequencing and other means to further identify the substrates of the zDHHC family, and the structures of the relevant zDHHC enzymes and corresponding substrates can be determined by X-ray crystallography or cryo-electron microscopy (cryo-EM), which can lead to the clarification of the molecular mechanisms by which palmitoylation modulates the function of the nervous system. Different neurological disorders arise as a result of mutations, aberrant expression, or direct or indirect aberrant regulation of zDHHCs. There are 23 members of the zDHHC family, of which several have been found to be associated with neurological disorders, and the presence of abnormalities in different zDHHCs increases the likelihood of neurological disorders. Using zDHHCs in the diagnosis and treatment of neurological diseases is a direction that needs to be investigated in the future, and new ideas and methods that restore zDHHC functioning in neurological diseases can be explored from the following using the fol-lowing three aspects: 1) Focus on posttranslational modifications that modify zDHHCs and regulate zDHHC pal-mitoylation by modulating the corresponding posttranslational modifications. 2) Develop novel high-throughput assays for the discovery of zDHHC-associated inhibitors. However, the role of zDHHC in the nervous system needs to be accounted for when proceeding with the clinical application of inhibitors for zDHHC-associated diseases. 3) Ma-nipulate specific substrates for zDHHC conversion to alter the state of individual protein palmitoylation.
